# Monitoring and manipulating autophagy in potato psyllids: impacts on accumulation and transmission of “*Candidatus* Liberibacter solanacearum” haplotypes A and B

**DOI:** 10.1128/spectrum.02068-25

**Published:** 2025-08-19

**Authors:** Junepyo Oh, Mingyung Lee, Cecilia Tamborindeguy

**Affiliations:** 1 Department of Entomology, Texas A&M University 171804, College Station, Texas, USA; 2 Department of Animal Science, Texas A&M University 199048, College Station, Texas, USA; USDA-ARS San Joaquin Valley Agricultural Sciences Center, Parlier, California, USA

**Keywords:** *Bactericera cockerelli*, "*Candidatus* Liberibacter solanacearum", rapamycin, autophagy

## Abstract

“*Candidatus* Liberibacter solanacearum” (Lso) is a highly destructive plant pathogen within the alpha-proteobacteria group. Multiple Lso haplotypes occur worldwide, each uniquely associated with a specific psyllid vector. Haplotypes A and B, found in the United States, cause serious damage to solanaceous crops and are transmitted by *Bactericera cockerelli*, known as the potato psyllid in a circulative and persistent manner. The psyllid gut is the first organ encountered by Lso and may act as a barrier to its transmission; however, the immune response of the gut to Lso infection remains largely uncharacterized. In this study, we examined autophagic responses in the gut of potato psyllids at early, mid, and late infection stages. Based on gene expression analyses, microscopic observations, and Western blotting, we found no clear evidence of autophagy induction despite Lso infection. Nevertheless, during mid and late phases of LsoB infection, we observed downregulation of the mechanistic target of rapamycin and decreased accumulation of ATG8-II. Notably, inducing autophagy with rapamycin significantly reduced LsoA titers in the psyllid guts after a 5-day acquisition access period and lowered transmission efficiency, while LsoB remained unaffected. These findings suggest that modulating autophagy in the gut of potato psyllids could be a promising strategy to limit LsoA acquisition and transmission, while highlighting LsoA and LsoB possess distinct molecular regulatory mechanisms in the psyllid gut.

**IMPORTANCE:**

Liberibacters are devastating plant pathogens transmitted by psyllids. Because these bacteria are fastidious, the study of the molecular mechanisms involved in plant infection and transmission is difficult. Here, we determined that inducing autophagy in the potato psyllid can affect the acquisition and transmission of Lso haplotype A but not haplotype B. Comparing the host and vector responses to different liberibacters can help identify their transmission and infection mechanisms and find targets to disrupt these processes.

## INTRODUCTION

Autophagy is a lysosomal breakdown process for superfluous and/or damaged cellular components that safeguard cells from nutritional or cellular stress (1, 2). During the autophagic response, the cell compartments targeted for breakdown are sequestered in double-membrane vesicles, the autophagosomes. After they are formed, the autophagosomes develop into autolysosomes through the fusion of lysosomes for degradation and recycling (3). Autophagy is also a defensive mechanism (4, 5). Indeed, insects can activate autophagy as part of their defense responses to stop a pathogen invasion. A number of studies have revealed that vector-borne plant viruses hijack autophagy in hemipteran insects (6–11).

“*Candidatus* Liberibacter solanacearum” (Lso) is a gram-negative, phloem-limited, and unculturable bacterium (12). More than 10 haplotypes have been described in the world based on SSR, 16S, or MLST sequences (13–17). Each haplotype has specific associations with its insect vector and host plants. Among them, haplotypes A and B are transmitted by *Bactericera cockerelli* (Šulc; Hemiptera: Triozidae), known as the potato psyllid or the tomato psyllid, and cause zebra chip disease, a fatal disease that affects potatoes in the Americas and severely damages other solanaceous crops (18). Both haplotypes are persistently transmitted by potato psyllids, and the gut is the initial organ invaded by Lso, serving as a barrier to its transmission (19). However, how Lso overcomes the barrier, that is, enters, propagates, and exits midgut cells while overcoming psyllid immunity for successful transmission, is unclear. Therefore, understanding the molecular interactions between psyllids and Lso in the gut is necessary to unravel the mechanisms of Lso acquisition or transmission.

Recently, the involvement of autophagy in the Liberibacter-psyllid complex was reported. For instance, LsoD induced autophagy in the gut of carrot psyllids, and the induction of autophagy decreased LsoD titer in the gut of psyllids (20, 21). This indicates that autophagy is part of the immune response activated in the gut against LsoD infection. In contrast, “*Candidatus* Liberibacter asiaticus” (CLas) could hijack the autophagic pathway in the Asian citrus psyllid to complete its life cycle in the midgut cells (22). Therefore, in the case of CLas, hijacking autophagy would benefit the pathogen. In potato psyllids, our prior transcriptomic research revealed the potential induction of autophagy in response to LsoB acquisition (23). In an effort to evaluate whether autophagy was occurring in response to LsoA or LsoB, we annotated 19 autophagy-related genes (ATGs) encoded by the potato psyllid and evaluated their expression (24). We determined that a majority of the ATGs potentially involved in the steps previous to the phagophore formation were upregulated in the gut of psyllids upon LsoA acquisition, while fewer genes were regulated in response to LsoB (24). Based on these conflicting results, it was necessary to evaluate whether these transcriptional changes in the potato psyllid gut led to autophagy or whether these two Lso haplotypes could modulate this pathway differently. Because autophagy is a multi-step process and ATG expression alone is insufficient to infer pathway activation or suppression, we have since validated the tools to evaluate autophagic responses in the gut of potato psyllids (25).

The present study investigated the involvement of autophagy in potato psyllids in response to LsoA and LsoB infection (24). We first compared the accumulation of LsoA and LsoB in the gut of potato psyllids after 1-, 5-, and 10-day acquisition access periods (AAPs), based on our previous study, these times correspond to key different events in the acquisition of the two haplotypes (19): 1-day AAP is an early stage of the gut infection for LsoA and LsoB; 5-day AAP represents the propagation stage for both haplotypes, LsoA titer is slowly increasing while LsoB titer is reaching its plateau, and 10-day AAP corresponds to a late time in the interaction between LsoB and the psyllid gut. LsoB titer already plateaued, while LsoA titer still increases and is close to reaching the plateau. Then, we evaluated the autophagic activity in the gut of psyllids in response to LsoA and LsoB after 1-, 5-, and 10-day AAPs, by combining gene expression analysis, microscopy to visualize lysosomes and autolysosomes, and western blotting to assess the autophagic flux, which reflects the autophagic degradation activity. Finally, using rapamycin, an autophagy inducer in psyllids, we tested the role of autophagy in Lso acquisition and transmission. This study contributes to a better understanding of the role of cellular immunity against the intracellular pathogen Lso.

## RESULTS

### Accumulation of LsoA and LsoB

**Fig 1:**
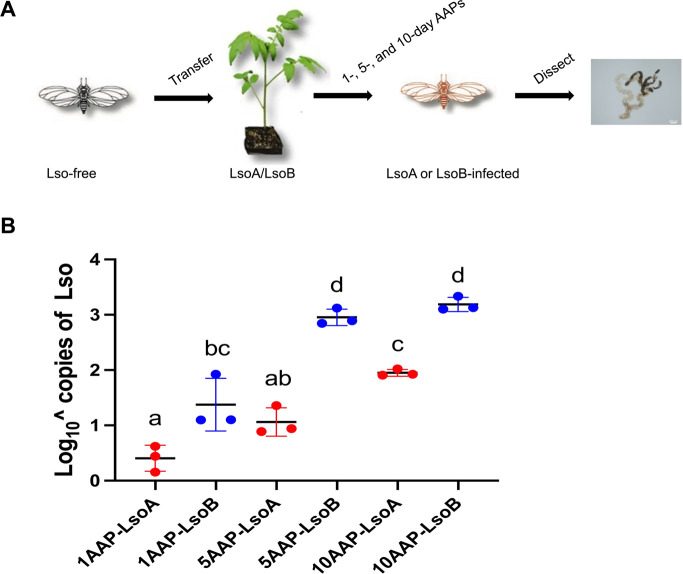
Quantification of Lso in the gut of potato psyllids following acquisition. (**A**) Adult female psyllids were transferred to LsoA‐ or LsoB-infected tomato plants for 1-, 5-, and 10-day AAPs. The pooled guts from 30 insects were used for Lso quantification. (**B**) Quantification of Lso copies in the gut of potato psyllid following Lso acquisition. The bars represent the Log_10_ copies of LsoA (red) and LsoB (blue) in pools of 30 guts following a 1-, 5-, and 10-day AAPs. The data represent the means ± SD of three biological replicates. Two-way ANOVA followed by Tukey’s *post hoc* test was used to evaluate the effects of the two independent variables (haplotypes and AAPs) and their interaction. Different letters indicate statistically significant differences between groups (*P* < 0.05); groups sharing the same letter are not significantly different.

Lso titer was measured in the gut of psyllids after 1-, 5-, and 10-day AAPs (Fig. 1A). A two-way analysis of variance (ANOVA) was performed to compare the effects of haplotype and AAP on the Lso accumulation in the gut of adult psyllids. The results showed a significant effect of the haplotype [*F* (1, 12) = 129.0921, *P* < 0.0001] and the AAP [*F* (2, 12) = 67.3910, *P* < 0.0001]. The interaction between the two variables was also significant [*F* (2, 12) = 5.2032, *P* = 0.0236]. Both LsoA and LsoB were detectable in pools of 30 guts of adult potato psyllids after a 1-day AAP, and LsoB titer was significantly higher than LsoA (Fig. 1B). LsoB titer rapidly increased to an average of Log_10_ 2.95 copies after 5 days and Log_10_ 3.18 copies after a 10-day AAP. This result indicated that LsoB titer had plateaued by the 5-day AAP. LsoA titer also increased over time, but it reached Log_10_ 1.06 and Log_10_ 1.95 after 5- and 10-day AAPs, respectively. LsoB titer was significantly higher than LsoA at each AAP (Fig. 1B). These results confirmed previous reports of differences in LsoA and LsoB accumulation in the gut of adult psyllids (19). Briefly, LsoB titers increased fast, plateauing after 6 days, while LsoA titers increased at a slower pace, plateauing at the same level as LsoB after a 12-day AAP (19).

### Lack of evidence of autophagy in the gut of potato psyllids upon Lso infection

**Fig 2:**
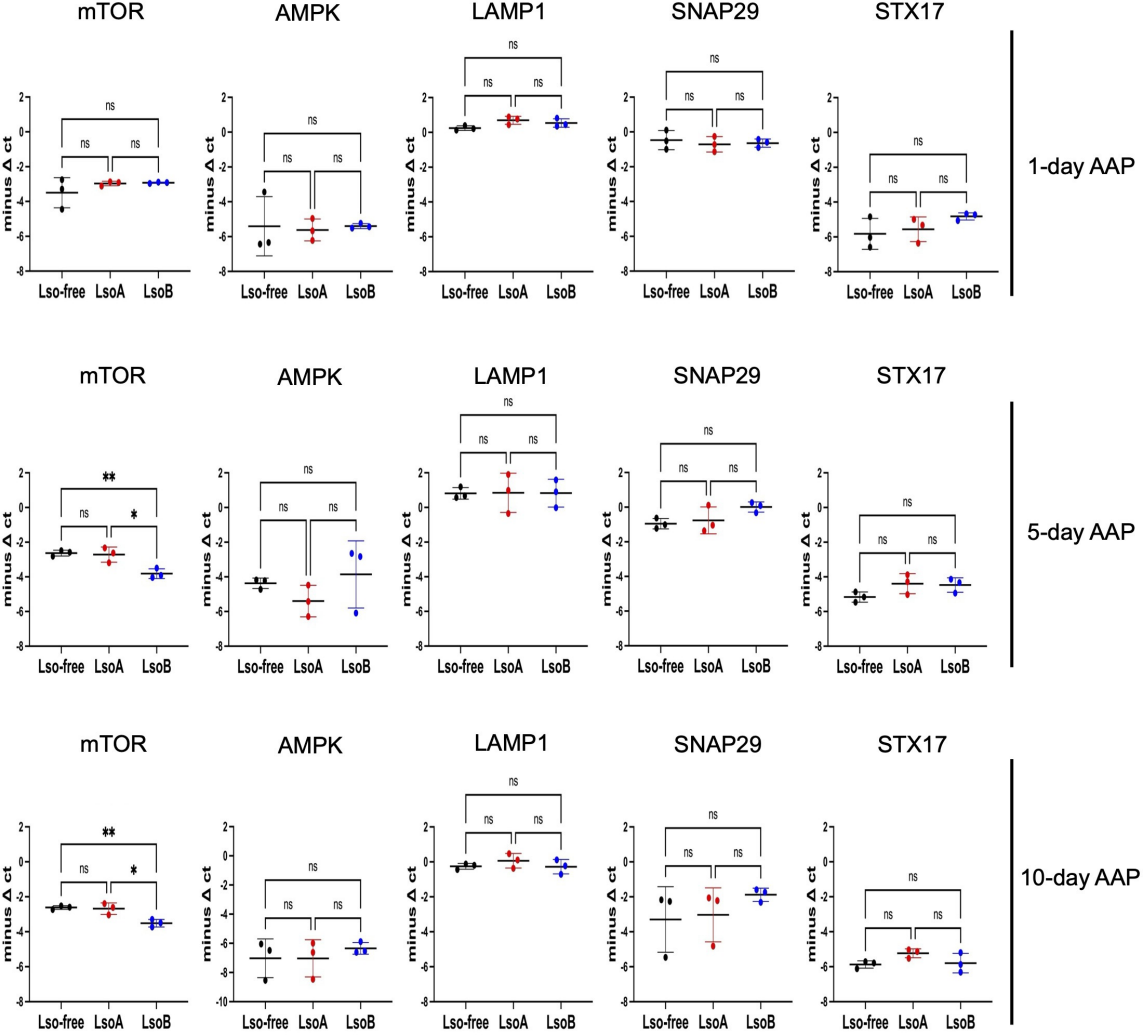
Relative expression of the genes involved in autophagic responses in the gut of potato psyllids after 1-, 5-, and 10-day AAPs on LsoA or LsoB-infected tomato plants. All values are represented as mean ± SD. One-way ANOVA followed by Tukey’s *post hoc* test was used to determine statistical differences among groups. Statistically significant differences are indicated as follows: * for *P* < 0.05 and ** for *P* < 0.01; “ns” indicates a non-significant difference.

Autophagy is an immune response that could be linked to the difference between LsoA and LsoB accumulation. Previously, we had identified differential expression of ATGs in the gut of adult psyllids following LsoA or LsoB acquisition (24). To extend this finding beyond the canonical ATGs, we further investigated the expression of five additional autophagy markers that represent upstream regulation, autophagosome formation, and lysosomal activity in the gut of psyllids. The evaluation was performed at a 1-day AAP, the early acquisition stage, 5-day AAP when LsoB titer is reaching its plateau, and 10-day AAP when LsoA titer is reaching its plateau. Only mechanistic target of rapamycin (mTOR), a negative regulator of autophagy, was regulated after a 5- and 10-day AAP on LsoB-infected plants (Fig. 2). Overall, these results suggest that autophagy might not be induced in response to LsoA, while in response to LsoB infection, mTOR is downregulated, potentially resulting in autophagy induction.

**Fig 3:**
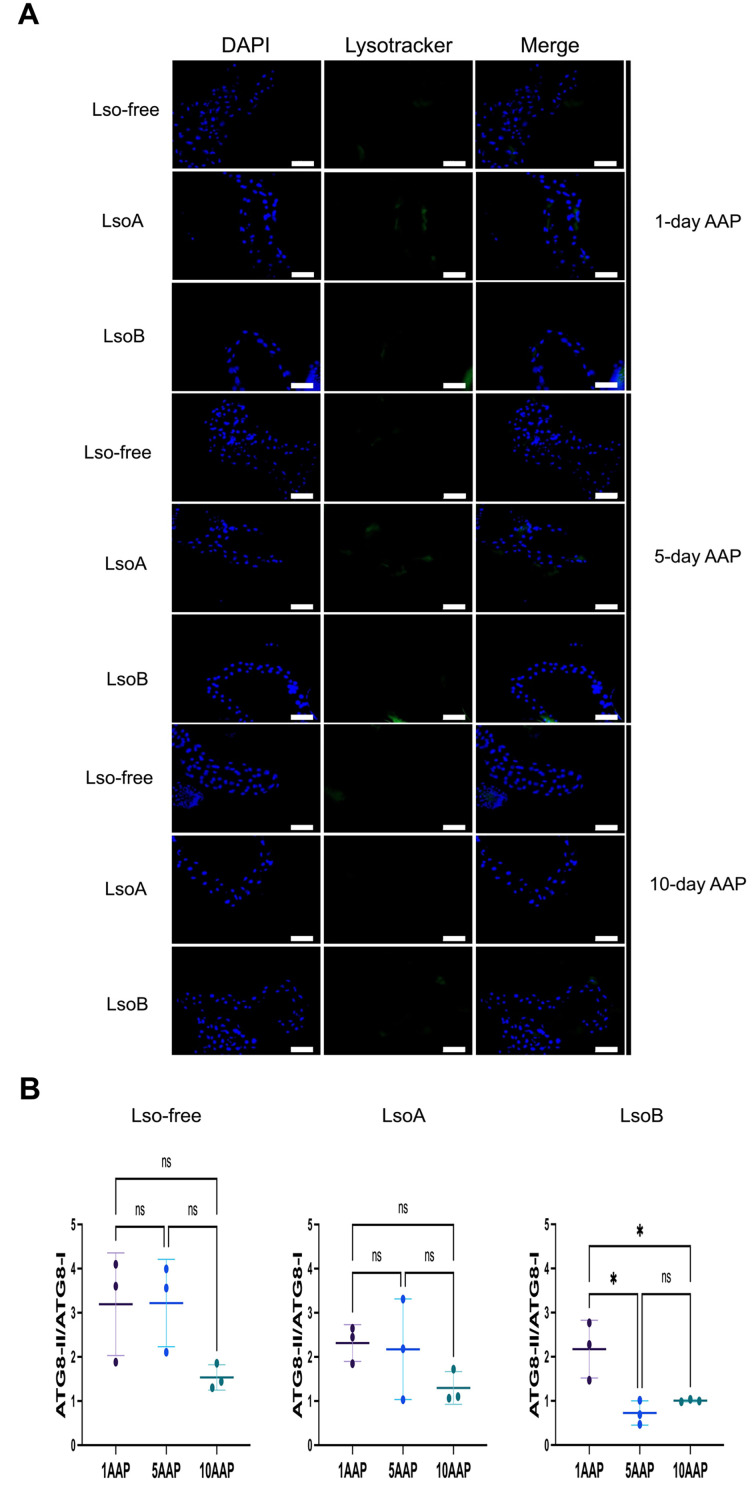
Autophagy evaluation in the gut of psyllids after 1-, 5-, and 10-day AAPs on LsoA- and LsoB-infected plants. (**A**) Staining of lysosomes and autolysosomes. The guts were stained with Lysotracker Green DND-26 staining (lysosomes and autolysosomes, green) and DAPI (nuclei, blue). At least 15 guts per AAP and Lso haplotype were observed. Scale bar = 100 µm (**B**) Measurement of ATG8-II/ ATG8-I following Western blot using ImageJ software (images and values are shown in Fig. S1B; Table S1). All values are represented as mean ± SD. Statistical differences among groups were evaluated using one-way ANOVA with Tukey’s *post hoc* test. Significance is denoted as follows: * for *P* < 0.05; “ns” indicates that the difference is not statistically significant.

We also investigated if autophagosomes and lysosomes could be observed in the gut of psyllids using Lysotracker. Psyllid guts were stained with Lysotracker after 1-, 5-, and 10-day AAPs on LsoA- or LsoB-infected plants. Our results showed no changes in the Lysotracker signals, indicating no changes in the lysosome and autolysosome quantity upon LsoA or LsoB infection compared to Lso-free in the monitored time points (Fig. 3A). In contrast, rapamycin-treated psyllid guts (positive control) showed signals of lysosomes and autolysosomes (Fig. S1A).

To further validate the absence of autophagy in response to LsoA or LsoB acquisition, we evaluated the expression of ATG8-I and ATG8-II by Western blot analyzes (Fig. S1B). The results of the analysis in the guts of psyllids after 1-, 5-, and 10-day AAPs on Lso-free, LsoA- or LsoB-infected tomato plants showed a significant decrease in the ATG8-II/ATG8-I ratio in the gut of psyllids after 5- and 10-day AAPs on LsoB-infected plants compared to a 1-day AAP. No statistically significant differences were found in the gut of psyllids on Lso-free or LsoA-infected tomato plants over time (Fig. 3B). These results are consistent with the Lysotracker results and indicate the absence of induced autophagy following the acquisition of LsoA or LsoB. The decrease in ATG8-II/ATG8-I after 5- and 10-day APPs on LsoB-infected plants could signify that Lso is suppressing autophagy at these time points.

### Effects of induction of autophagy on Lso accumulation

**Fig 4:**
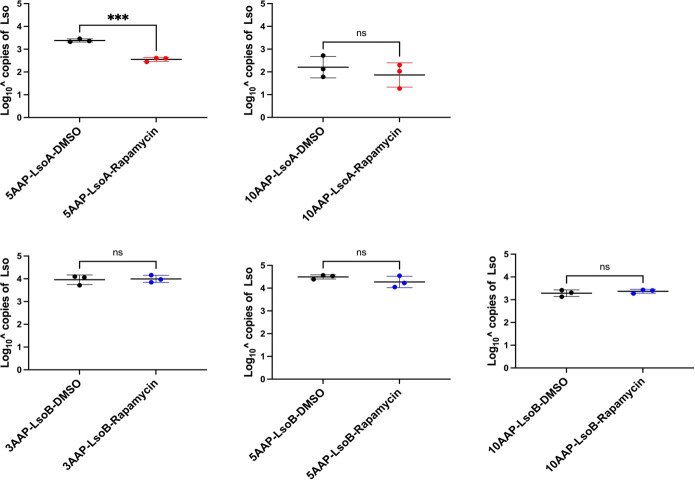
Quantification of LsoA (red) and LsoB (blue) in the gut of psyllids after feeding on rapamycin to induce autophagy following 5- and 10-day AAPs for LsoA and 3-, 5-, and 10-day AAPs for LsoB. All values are represented as mean ± SD. Statistical differences between groups were determined using Student’s *t*-test. Three asterisks indicate *P* < 0.001, while “ns” denotes a non-significant difference.

Because autophagic responses were not significantly induced in the gut of psyllids after 1-, 5-, or 10-day AAPs, while LsoB may reduce autophagy, we hypothesized that Lso could suppress autophagy in the gut of psyllids. To test this hypothesis, we first used rapamycin to induce autophagy in the psyllid gut as previously described in Oh and Tamborindeguy (25) and examined Lso accumulation. For this evaluation, psyllids were allowed different AAPs followed by 24 h on a diet containing or not rapamycin. Treatment with rapamycin decreased the accumulation of LsoA in the gut of psyllids only after a 5-day AAP (Fig. 4). The LsoB titer remained similar in the gut of rapamycin-treated insects similar to the control insects at all tested time points (Fig. 4). Because LsoB accumulates fast in the gut of psyllids, we also included a shorter AAP for this haplotype, before the plateau was reached; still, there was no effect of the rapamycin treatment on the LsoB titer.

### Effects of induction of autophagy in LsoA transmission

**Fig 5:**
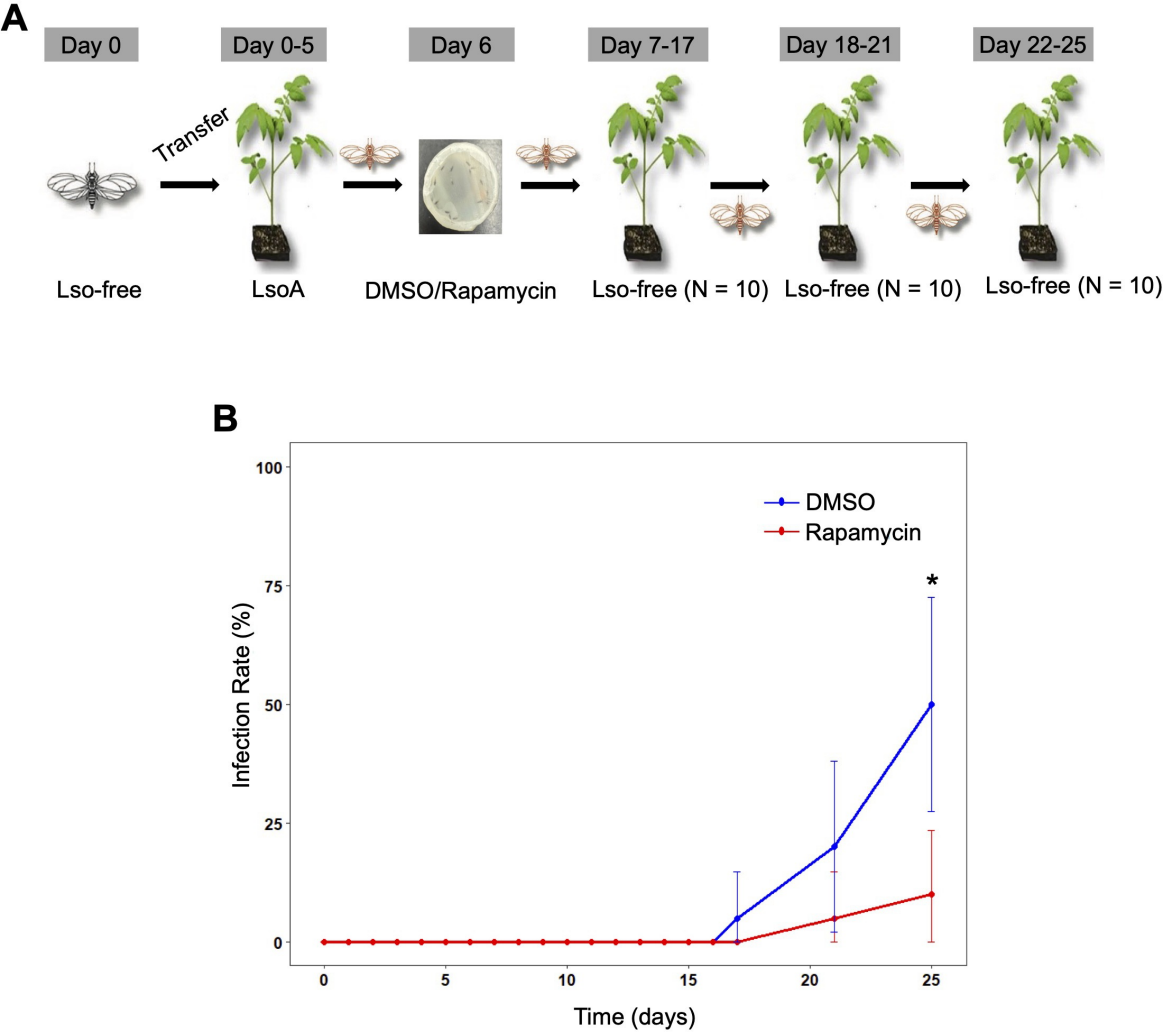
Sequential transmission of LsoA to tomato plants by potato psyllids. Female adults were given a 5-day AAP on LsoA-infected tomato plants and fed a rapamycin- or DMSO (control)-containing diet for 1 day. Then, groups of five psyllids were transferred to 10 non-infected recipient tomato plants for an 11-day inoculation access period (IAP). Then, each group was sequentially transferred to a new uninfected recipient plant every 4 days (IAP). LsoA transmission was evaluated by PCR. (**A**) Schematic representation of the assay. (**B**) Percentage of infected plants. Data represent mean infection rate (%) ± 95% Wilson confidence intervals (CI) for each group. * indicates *P* < 0.05.

Because the induction of autophagy only affected LsoA titer after a 5-day AAP, we hypothesized that the transmission of this haplotype could also be affected by the induction of autophagy. Therefore, we induced autophagy using 1 day of feeding on rapamycin after a 5-AAP of LsoA. Then, we performed sequential inoculation of tomato plants (Fig. 5A). The plant infection rate varied between the rapamycin-treated and DMSO-treated (control) groups throughout the experimental period (Fig. 5B; Table S2). In the DMSO group, the first inoculation was detected 17 days after the beginning of acquisition, and by day 25, 50% of the plants were infected in this treatment. However, the first infected plant was detected after day 21 in the rapamycin treatment and, by day 25, only 10% of the plants were infected (Table S2).

Statistical analysis using Fisher’s exact test indicated a significant difference in infection prevalence between the two groups on day 25 (*P* = 0.014, Table S3). The odds of infection in the rapamycin-treated group were approximately 11% of those in the DMSO-treated group, indicating a substantial reduction in the transmission efficiency following rapamycin treatment. On days 17 and 21, no statistically significant differences in infection rates were detected between the two groups (Table S3).

## DISCUSSION

Vector-borne plant diseases are highly destructive and present significant challenges for effective control and management. The complex interactions between insect vectors and plant pathogens complicate the development of novel management strategies for these diseases. One promising approach involves disrupting the molecular interactions that are critical for pathogen acquisition and transmission by the vector. As invaders, plant pathogens must overcome the insect vector’s immune responses, which act as a primary barrier against infection.

In the present study, we investigated autophagic responses in the gut of potato psyllids in response to Lso infection. To elucidate the role of autophagy in Lso infection, we first compared LsoA and LsoB accumulation in the gut of psyllids after 1-, 5-, and 10-day AAPs corresponding to different phases of the gut infection. Our results showed that both haplotypes were detectable as early as after a 1-day AAP. However, the two haplotypes exhibited different accumulation dynamics over time. LsoB increased more rapidly than LsoA, resulting in significantly higher titers at each time point. Moreover, LsoB titer had plateaued by the 5-day AAP, whereas LsoA did not reach the plateau, which suggests the possibility that LsoA titers would continue to increase at later time points. This observation confirmed the previous study that examined the accumulation of LsoA and LsoB in the gut of psyllids after 0–16 days of AAP (19). In that study, LsoB titers increased significantly, reaching a plateau at 6 days of AAP, whereas LsoA titers rose more slowly, plateauing at 12 days of AAP.

Different Lso accumulation patterns between LsoA and LsoB can be linked to their difference in pathogenicity. Both haplotypes reduced psyllid oviposition; however, LsoB also induced nymphal mortality (26). These findings suggest that LsoA and LsoB interact differently with psyllids, leading to distinct psyllid responses to infection. Indeed, different transcriptional regulations between two haplotypes and psyllids were found in a transcriptomic study (23).

The gut transcriptomic study revealed the upregulation of ATGs in response to LsoB (23), while more ATGs were upregulated in the gut of psyllids in response to LsoA (24). To evaluate the autophagic responses in the gut of psyllids in response to Lso infection, we tested the expression of the genes involved in the autophagic pathway. We focused on five key genes: mTOR, AMPK, LAMP, SNAP, and STX-17, hypothesizing differential regulation in response to Lso infection. Our results revealed that LsoB only significantly downregulated mTOR after 5- and 10-day AAPs, while LsoA did not affect the expression of the tested genes. mTOR is a negative regulator of autophagy, and inhibition of mTOR can induce autophagy (27). Thus, the persistent downregulation of mTOR in response to LsoB after 5- and 10-day AAPs suggests a potential induction of autophagy. These results were in line with the transcriptomic results. However, autophagy is not regulated by mTOR alone or at the transcriptional level (28). Therefore, further validation was required (29).

We next investigated whether autophagy was modulated in the gut of psyllids following different AAPs. The number of autophagosomes and lysosomes following Lso acquisition did not increase based on Lysotracker staining. The result of autophagic flux showed a significant reduction in ATG8-II/ATG8-I after 5- and 10-day AAPs on LsoB-infected plants. This result suggested that despite the gene expression data, LsoB infection might decrease the formation of autophagosomes after 5- and 10-day AAPs. Overall, there was no evidence of autophagy modulation in response to LsoA, while there could be a potential reduction of autophagy in response to LsoB. This result led us to propose the following hypotheses. First, Lso infection does not trigger stress responses and cellular immunity in the midgut cells. Alternatively, an autophagic response occurs but is not widespread enough to be detected using the chosen approaches or the responses are highly dynamic and occur at different time points than the ones selected for the analysis. Indeed, the induction of autophagy in carrot psyllids and Asian citrus psyllids against LsoD and CLas was tested in the insects from infected colonies (20–22). This suggests that chronic infection by these pathogens could activate autophagy. Third, LsoA and LsoB suppress their vector immune responses similar to other pathogens (30). Indeed, some pathogenic bacteria such as *Salmonella* create a favorable intracellular environment by suppressing autophagy (31).

Next, we induced autophagy using rapamycin and examined its effects on Lso accumulation and transmission to test our third hypothesis. Notably, LsoA titer decreased following a 5-day AAP with a subsequent 1-day feeding on rapamycin. However, no significant change in LsoA titer was observed after a 10-day AAP prior to the rapamycin treatment. These results suggest that LsoA may employ strategies to evade the host cell’s autophagic pathway. Furthermore, the absence of effect after a 10-day AAP with a subsequent 1-day feeding on rapamycin suggests that LsoA might establish long-term survival mechanisms within the cell, potentially enhancing its ability to evade autophagy at later stages of infection. In addition, we found that rapamycin-induced autophagy increased the latency period and reduced the transmission efficiency of LsoA. Specifically, the first detection of LsoA-infected plants by psyllids from the rapamycin-treated groups occurred 21 days post-acquisition, compared to 17 days post-acquisition in the control groups. Moreover, the transmission efficiency was lower in the rapamycin-treated groups. These findings suggest that LsoA may suppress autophagy in the psyllid gut during the mid-stage of infection, thereby enabling stable propagation within gut cells. In contrast, the induction of autophagy by rapamycin had no discernible impact on LsoB titer. This indicates that LsoB may either resist autophagy induction by rapamycin by decreasing the formation of autophagosomes or utilize a survival strategy independent of the autophagy pathway. These observations support the hypothesis that LsoA and LsoB employ distinct mechanisms to infect the vector. Interestingly, our results for LsoA align with findings for LsoD, which showed rapamycin-induced autophagy decreased LsoD titer in carrot psyllids (20). Since LsoA shares greater genomic similarity with LsoD than with LsoB (32), it is plausible that LsoA and LsoD exhibit similar biological interactions with their insect vectors, despite associating with different host plants and insect vectors. However, the mechanisms by which Lso suppresses autophagy in the psyllid gut remain unclear and require further investigation.

In summary, our study monitored and manipulated autophagic responses in the gut of psyllids in response to LsoA and LsoB infection. We did not find evidence of autophagic responses in the gut of psyllids following LsoA or LsoB acquisition, and chemically induced autophagy reduced LsoA accumulation after a 5-day AAP. This reduction in LsoA accumulation was accompanied by a longer latency and a reduced transmission efficiency. However, the induction of autophagy by rapamycin had no impact on LsoB accumulation. Our study expanded our knowledge of the molecular interactions between two Lso haplotypes and psyllids and supported the notion that different Lso haplotypes deploy specific strategies to infect their vectors leading to differences in pathogenicity, accumulation, and transmission by psyllids.

## MATERIALS AND METHODS

### Plant and insect colonies

Tomato (*Solanum lycopersicum* L. ‘Moneymaker’; Thompson and Morgan Inc., Jackson Township, NJ, USA) seeds were sown in pots containing Sun Gro Sunshine LP5 mix (Bellevue, WA, USA). The obtained plants were fertilized with Miracle-Gro Water-Soluble Tomato Plant Food at the label rate (18-18-21 NPK; Scotts Miracle-Gro Company, Marysville, OH, USA).

Insect-proof cages (24 by 13.5 by 13.5 cm; BioQuip, Compton, CA, USA) were used to keep psyllid colonies harboring Lso-free, LsoA, and LsoB on tomato plants. Each psyllid colony was kept separate and placed under a photoperiod consisting of 16 h of light and 8 h of darkness at room temperature (22 ± 2°C). Periodically, PCR was used for Lso detection in each colony. The LsoF/012 primers were used for Lso detection and the SSR1 primers for haplotype determination (17, 33).

### Psyllid exposure to Lso and midgut dissection

Fifty Lso-free adult female psyllids, approximately 7 days old, were transferred to tomato plants infected with LsoA or LsoB or to uninfected plants. The midguts were dissected after 1-, 5-, and 10-day AAPs under the Leica EZ4W0037 stereomicroscope (Leica Microsystems, Wetzlar, Germany) as described in reference 34. There were three biological replicates for each haplotype and AAP.

### Quantification of Lso

DNA from pools of 30 psyllid guts was extracted following the procedure described in reference 35. Lso titer was measured by qPCR using the LsoF and HLBR primers (33, 36). As an internal control, the 28s rDNA of psyllids was amplified (35). The QuantStudio 6 Flex Real-Time PCR System (Applied Biosystems, Foster City, CA, USA) was used to conduct the qPCR. All qPCRs were performed using a PowerUp SYBR Green Master Mix (Applied Biosystems, Waltham, MA, USA) following the manufacturer’s instructions. Each technical replicate was composed of 1× SYBR master mix, 0.25 µL of each primer at 250 nM, and 25 ng of DNA. Nuclease-free water was used to modify the total volume of each reaction to 10 µL. The qPCR program conducted was 40 cycles at 95°C for 2 min, 95°C for 5 s per cycle, and 60°C for 30 s in a QuantStudio 6 Flex Real-Time PCR System (Thermo Fisher Scientific, Waltham, MA, USA). Each experiment contained a negative control and two technical replicates per reaction.

A standard curve for the quantification of Lso was generated using a plasmid containing the Lso 16S rDNA as previously described by Levy et al. (37). Absolute Ct values of the Lso 16S rDNA were then used to determine Lso titer in psyllid guts. Three biological replicates were analyzed.

### RNA extraction, cDNA synthesis, and RT-qPCR

RNA from pools of 30 psyllid guts was purified using the RNeasy Mini Kit (Qiagen, Valencia, CA, USA) and treated with Turbo DNase (Ambion, Austin, TX, USA). cDNA was synthesized using a Verso cDNA Synthesis Kit (Thermo, Waltham, MA, USA) from 200 ng of RNA and anchored oligo (dT) primers as described in the kit’s manual. Then, the cDNA was diluted by adding 60 µL of water for qPCR analysis. For each experiment, three biological replicates (pools) were analyzed.

The expression of five genes involved in the autophagic responses was evaluated by quantitative PCR (qPCR). The tested genes included the mTOR, AMP-activated protein kinase (AMPK), lysosomal-associated membrane protein 1 (LAMP1), synaptosomal-associated protein 29 (SNAP29), and syntaxin 17 (STX17). mTOR is a protein kinase that inhibits autophagy induction through the phosphorylation of autophagy-related complex components (38) whereas AMPK promotes autophagy by phosphorylating autophagy-related proteins (39). LAMP1 is a key component of the lysosomal membrane, facilitating the fusion of autophagosomes with lysosomes (40). SNAP29 also mediates membrane fusion between autophagosomes and lysosomes (41). STX17 is a SNARE (soluble N-ethylmaleimide-sensitive factor attachment protein receptor) protein that helps autophagosome fusion (42). The primers for qPCR are listed in Table S4. Each reaction included 2 µL of diluted cDNA, 250 nM of each primer, and 5 µL of PowerUp SYBR Green Master; nuclease-free water was used to adjust the volume to 10 µL. The qPCR program was 95°C for 2 min followed by 40 cycles of 95°C for 5 s and 60°C for 30 s Three technical replicates were performed for each reaction, and negative controls (no cDNA) were included in each run. The qPCRs were run in a QuantStudio 6 Flex Real-Time PCR System (Thermo Fisher Scientific). The relative expression of the candidate genes was calculated using the minus ∆ Ct method with two reference genes ribosomal protein subunit 18 (GenBank KT279693) and elongation factor-1a (GenBank KT185020) (43) and the Lso-free treatment as a reference.

### Lysotracker green DND-26 staining

To visualize the induction of autophagy in the potato psyllid gut following a 1-, 5-, and 10-day AAP on Lso-free, LsoA-, and LsoB-infected plants, the gut of adult female psyllids was dissected as previously described and transferred to 50 µL dye solution containing 1 µM of Lysotracker Green DND-26 (Invitrogen, Carlsbad, CA, USA) and 10 µg/mL of DAPI (Sigma-Aldrich, St. Louis, MO, USA). The guts were incubated in the dye solution as described in Oh and Tamborindeguy (25). The guts were mounted using a 1× phosphate-buffered saline (1× PBS) solution (Sigma-Aldrich) and observed immediately under a fluorescent microscope (Axio Imager A1 microscope, Carl Zeiss Microscopy, White Plains, NY, USA). Three replicates were analyzed, each containing at least fifteen guts.

We also used the autophagy inducer rapamycin by feeding, as a positive control as described in Oh and Tamborindeguy (25). Briefly, rapamycin at a concentration of 10 µM was added to the diet containing 15% (wt:vol) sucrose and 1× PBS solution. A minimum of 30 female psyllids were placed in plastic feeding chambers (*h* = 2 cm, Φ = 3 cm), which were covered with two sheets of Parafilm with 100 µL of the liquid diet between two layers. After 24 h of feeding, the guts from the rapamycin-treated psyllids were dissected and stained with Lysotracker Green DND-26 as described above.

### Protein extraction and western blotting

The autophagic flux is characterized by ATG8 lipidation, which is the conversion of ATG8-I to ATG8-II. These proteins are necessary for the elongation and maturation of the autophagosomes, and the conversion ratio reflects the amount of autophagosomes that subsequently fuse with lysosomes for degradation. Guts of psyllids following a 1-, 5-, and 10-day AAP on Lso-free, LsoA-, and LsoB-infected plants were dissected and pooled as previously (each pool had 20 guts). Proteins were purified using the RIPA buffer (Invitrogen) supplemented with one tablet of Protease Inhibitor Mini Tablets (Thermo Fisher Scientific). Ten micrograms of proteins was mixed with 4× SDS sample buffer and separated on a 4–12% Bis-Tris NuPage gel (Invitrogen). Then, following standard blotting procedures, the proteins were transferred to an Immobilon-P PVDF membrane (Millipore-Sigma, Burlington, MA, USA) and visualized using a Pierce Reversible Protein Stain Kit for PVDF Membranes (Thermo Fisher Scientific). The membrane was blocked with 5% dry milk in the TBST buffer at room temperature for 1 h, followed by incubation with the primary antibody anti-GABARAP + GABARAPL1 + GABARAPL2 (ATG8) (ab109364; Abcam, Cambridge, United Kingdom) at 4°C overnight. After washing off the primary antibody, the blot was incubated with an anti-rabbit IgG secondary antibody (Sigma-Aldrich) at room temperature for 1 h. The SuperSignal West Pico substrate (Invitrogen) was used to detect the signal on an iBright 1500 imaging system (Thermo Fisher Scientific). The ImageJ software was used to analyze and quantify the signal intensity, and the autophagic flux was calculated using the ratio of the ATG8-II/ATG8-I. Three independent replicates were analyzed.

### Induction of autophagy and its effects on Lso acquisition and transmission

To evaluate the effects of autophagy induction on the accumulation of Lso in the psyllid gut, we used rapamycin as an autophagy inducer (25). Fifty Lso-free adult female psyllids were transferred to LsoA- or LsoB-infected tomato plants for 3- (LsoB only), 5-, and 10-day AAPs. These time points were chosen to evaluate the effect of autophagy at different time points in the invasion process; thus, because LsoB titer increases fast, an earlier time point was also tested for this haplotype. Following the AAP, psyllids were collected and placed in plastic feeding chambers (*h* = 2 cm, Φ = 3 cm) covered by two sheets of Parafilm with 100 µL of the liquid diet for 24 h. The liquid diet consisted of a sterilized solution of 15% (wt:vol) sucrose and 1× PBS (Sigma-Aldrich) with rapamycin dissolved in dimethyl sulfoxide (DMSO) at a concentration of 10 µM or with an equivalent amount of DMSO (control). Guts were then dissected and pooled (30 guts per pool) for Lso quantification. Three independent replicates were analyzed.

The effects of autophagy induction by rapamycin on the transmission of LsoA were evaluated: Lso-free adult females were allowed a 5-day AAP on LsoA-infected tomato plants followed by 24 h feeding on the rapamycin or DMSO-containing diets as described above. Groups of five rapamycin- and DMSO-fed psyllids were transferred to ten 4-week-old healthy tomato plants for the 11-day latent period for Lso transmission (19). After this latent period, every 4 days (inoculation access period [IAP]), each group of psyllids was sequentially moved to a new non-infected recipient tomato plant (10 plants per set). The additional psyllids were maintained on a non-infected tomato plant as a backup. Before each transfer, psyllid mortality was recorded and psyllids were added from the backup set to have five psyllids in each transfer. The sequential transmission ended on day 25 due to high psyllid mortality. A total of three transfer rounds were possible. The plants were evaluated for Lso infection two times, at weeks 4 and 6 post-inoculation, as previously described. This experiment was conducted twice independently.

### Data analysis

The data were analyzed using R version 4.4.2 (R Core Team, Vienna, Austria) and GraphPad Prism 9.5.1 Software (GraphPad Software, San Diego, CA, USA). Also, quantification data were subjected to a log 10-transformation for analysis to ensure normality. The Shapiro-Wilk test was used to evaluate the normal distribution of residuals, and Levene’s test was employed to verify the homogeneity of variance. The data were subjected to a two-way ANOVA with Lso haplotype and AAPs as independent variables, followed by Tukey’s post hoc test.

The gene expression data and the autophagic flux following Lso infection were analyzed by one-way ANOVA with Tukey’s post hoc test. Also, quantification following rapamycin treatment was compared using Student’s *t*-tests. For the transmission assays, Fisher’s exact test was used to assess the association between treatments and infection status. This test was chosen over the chi-square test due to the small sample size in certain groups, ensuring an accurate *P* value estimation without reliance on asymptotic approximations. Fisher’s exact test was conducted using the fisher.test() function in R, which computes an exact *P* value based on the hypergeometric distribution.

The odds ratio (OR) was calculated to quantify the relative likelihood of infection between the treatment groups. The OR was computed using the formula:


$$OR=\frac{a\times d}{b\times c}$$

where *a* and *b* represent the number of infected and non-infected samples in the rapamycin treatment group, respectively, and *c* and *d* correspond to the infected and non-infected counts in the DMSO control group.

To estimate the 95% confidence interval for the odds ratio, the profile likelihood method was employed, as implemented in fisher.test(). Unlike the Wald method, which relies on asymptotic normality, the profile likelihood method provides a more reliable confidence interval for small sample sizes by adjusting the likelihood function directly. This ensures that the estimated range accurately reflects the uncertainty in OR estimation.

For days where one treatment had no infections, resulting in an infinite OR, the lower bound of the confidence interval was reported, and the upper bound remained undefined (Inf). If the confidence interval included 1.0, the difference in infection risk between treatments was considered statistically non-significant. A *P*-value threshold of 0.05 was used to determine statistical significance.

## Data Availability

All data that support the findings of this study are provided in the paper.

## SUPPLEMENTAL MATERIAL

Supplemental material spectrum.02068-25-s0001.docx Fig. S1; Tables S1 to S4.

ASM does not own the copyrights to Supplemental Material that may be linked to, or accessed through, an article. The authors have granted ASM a non-exclusive, world-wide license to publish the Supplemental Material files. Please contact the corresponding author directly for reuse.
