# Supplementary material for: Monitoring and manipulating autophagy in potato psyllids: impacts on accumulation and transmission of “Candidatus Liberibacter solanacearum” haplotypes A and B
Source: Microbiol Spectr. 2025 Aug 19;13(10):e02068-25. doi: 10.1128/spectrum.02068-25 (PMC12502650; doi:10.1128/spectrum.02068-25)
Supplement: Supplemental material — Fig. S1; Tables S1 to S4. [file spectrum.02068-25-s0001.docx]

**Supplementary Figure and Tables Legend**

**Figure S1**. (A) Lysotracker staining of rapamycin fed psyllids was used as a positive control. (B) Western blotting of ATG8-I and ATG8-II for measuring autophagic flux. ImageJ software was used to quantify the expression of ATG8-I and ATG8-II (Table S1).

**Table S1.** Quantification of the expression of ATG8-I and ATG8-II (Fig. S1B) using ImageJ software.

**Table S2.** Infection rates with 95% confidence interval.

**Table S3.** Odds ratio and Fisher's exact test *P*-value.

**Table S4**. Primers in this study.


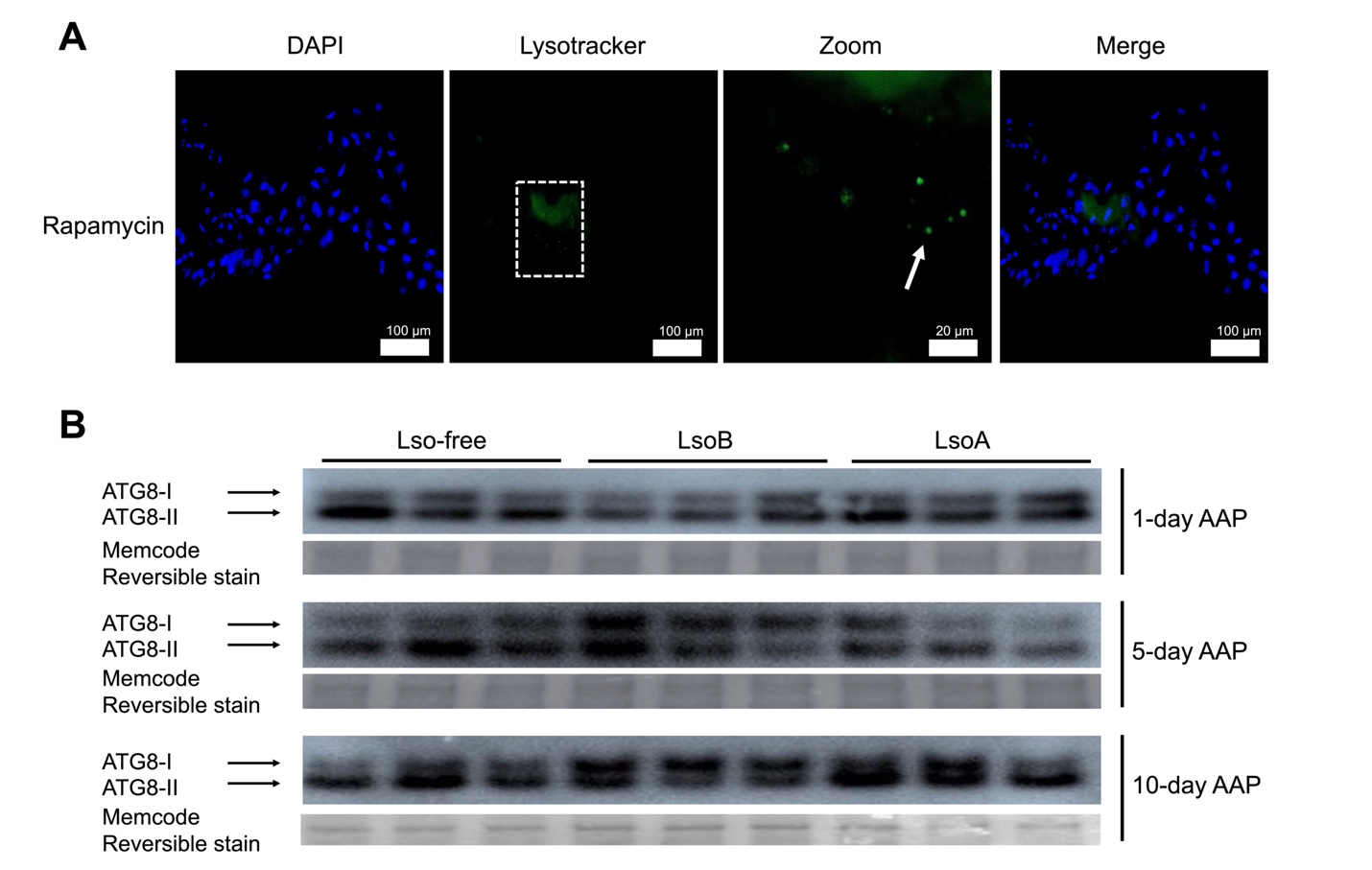
**Figure S1**

**Table S1.**

| **1AAP** | **ATG8-I** | **ATG8-II** | **Ratio**  **(ATG8-II/ATG8-I)** |
| --- | --- | --- | --- |
| JM1-1 | 18053.08 | 73966.37 | 4.09716 |
| JM1-2 | 24465.98 | 46040.4 | 1.881813 |
| JM1-3 | 15492.97 | 55836.27 | 3.603975 |
| JM3-1 | 13839.1 | 38295.14 | 2.76717 |
| JM3-2 | 14301.11 | 32587.34 | 2.278658 |
| JM3-3 | 27216.71 | 40016.07 | 1.470276 |
| JM4-1 | 25294.54 | 67005.14 | 2.648997 |
| JM4-2 | 23234.51 | 56870.83 | 2.447688 |
| JM4-3 | 41445.53 | 76653.49 | 1.849499 |
| **5AAP** | **ATG8-I** | **ATG8-II** | **Ratio**  **(ATG8-II/ATG8-I)** |
| JM1-1 | 10745.49 | 38245.35 | 3.559201 |
| JM1-2 | 12059.12 | 48178.63 | 3.995204 |
| JM1-3 | 15895.63 | 33471.2 | 2.105686 |
| JM3-1 | 48425.64 | 49211.37 | 1.016225 |
| JM3-2 | 37481.02 | 25914.26 | 0.691397 |
| JM3-3 | 27239.74 | 12776.36 | 0.469034 |
| JM4-1 | 28107.96 | 28928 | 1.029175 |
| JM4-2 | 8272.246 | 27368.31 | 3.30845 |
| JM4-3 | 7913.903 | 17288.27 | 2.184544 |
| **10AAP** | **ATG8-I** | **ATG8-II** | **Ratio**  **(ATG8-II/ATG8-I)** |
| JM1-1 | 15497.4 | 28776.7 | 1.856872 |
| JM1-2 | 26335.13 | 38054.91 | 1.445025 |
| JM1-3 | 23655.57 | 30848.01 | 1.304048 |
| JM3-1 | 39738.35 | 38956.96 | 0.980337 |
| JM3-2 | 36724.56 | 36641.58 | 0.997741 |
| JM3-3 | 35457.53 | 36720.5 | 1.035619 |
| JM4-1 | 37794.24 | 40234.41 | 1.064565 |
| JM4-2 | 35940.28 | 39623.32 | 1.102476 |
| JM4-3 | 22499 | 38806.88 | 1.724827 |

**Table S2**

| Day | Rapamycin | 95% CI Lower | 95% CI Upper | DMSO ^1^ | 95 % CI Lower | 95% CI Upper |
| --- | --- | --- | --- | --- | --- | --- |
| 17 | 0.00% | 0 | 0 | 5.00% | 0 | 14.8 |
| 21 | 5.00% | 0 | 14.8 | 20.00% | 2.0138 | 37.986 |
| 25 | 10.00% | 0 | 23.489 | 50.00% | 27.517 | 72.482 |

^1^ DMSO, dimethyl sulfoxide

**Table S3**

| Rapamycin vs. DMSO^1^ | Odds Ratio | 95% CI Lower | 95% CI Upper | *P*-value |
| --- | --- | --- | --- | --- |
| Day 17 | 0.000 | 0.000 | 39.001 | 1.000 |
| Day 21 | 0.218 | 0.004 | 2.501 | 0.342 |
| Day 25 | 0.117 | 0.010 | 0.709 | 0.014 |

^1^ DMSO, dimethyl sulfoxide

**Table S4**

| Primer | | |
| --- | --- | --- |
| Bc_mTOR_qF | CTCGCACGAAGAAATGCTCG | Gene expression |
| Bc_mTOR_qR | AGAAGGGACACCAGCCAATG |  |
| Bc_AMPK_qF | TATTACCGCGGGCAAGTCTG |  |
| Bc_AMPK_qR | TCCTAGGAGGACTTCCGGTG |  |
| Bc_LAMP1_qF | CTGACCAGAACCTGGACCAC |  |
| Bc_LAMP1_qR | GTGTATCGCGTCATCTGGGT |  |
| Bc_SNAP29_qF | CCAGCTCTCCGCGTAAGAAA |  |
| Bc_SNAP29_qR | GTCTTGAGAGTCCACCCAGC |  |
| Bc_STX17_qF | AAAATGGGGAGTGTTGCTGC |  |
| Bc_STX17_qR | CACATGCACAGGGTCTGGTA |  |
